# ﻿Solving the riddle of *Aspidium ameristoneuron* Fée, a misunderstood Cuban or Mexican species of *Ctenitis* (Dryopteridaceae) – Occasional Papers from the Herbarium Greuter, 7

**DOI:** 10.3897/phytokeys.265.173094

**Published:** 2025-11-04

**Authors:** Werner Greuter, Rosa Gloria Rankin Rodríguez

**Affiliations:** 1 Botanisches Museum, Berlin, Germany Botanisches Museum Berlin Germany; 2 Herbarium Mediterraneum, Orto Botanico, Palermo, Italy Herbarium Mediterraneum, Orto Botanico Palermo Italy; 3 Jardín Botánico Nacional, Universidad de La Habana, La Habana, Cuba Universidad de La Habana La Habana Cuba

**Keywords:** Cuba, Fée herbarium, ferns, Mexico, new combination, typification

## Abstract

The holotype of *Aspidium ameristoneuron*, the correct application of which has long been in doubt, has been identified in the herbarium RB in Rio de Janeiro. It was found to have been mislabeled, and its true origin is Mexico, not Cuba as stated in the protologue. The history of the labeling error is reconstructed, and the identity of *Aspidium ameristoneuron* with the species so far known as *Ctenitis salvinii* (here renamed *Ctenitis ameristoneuros***comb. nov.**) is demonstrated. The names and synonyms involved are typified.

## ﻿Introduction

The name *Aspidium ameristoneuron* (‘*ameristonevron*’) Fée, Mém. Foug. 8, Ic. Esp. Nouv.: 104. 1857 (for the spelling correction, see Turland et al. 2025: Art. 60.6, second sentence) has long been a puzzle to pteridologists. In the protologue (Fée 1857: 105), the original material for that name is referred to with a single word: “Cuba.”

In his “Index filicum”, Christensen (1905–1906: 251), following the opinion of Kunze, accepted the name *Aspidium ameristoneuros* (‘*ameristoneura*’) (Fée) C. Chr., with *Dryopteris grisebachii* (Baker) Kuntze in synonymy. However, he subsequently (Christensen 1920: 44) changed his opinion, writing: “[In 1905] I have followed Kuhn in identifying N[ephrodium]. grisebachii Bak. with A[spidium]. ameristoneuron Fée; now I have my serious doubts as to this being well founded. Fée’s description does not agree very well with N. grisebachii… Fée quotes no collector but the locality ‘Cuba’ only; probably he has here committed an error… I have no doubt … that the species of Fée is really Mexican.” The influential current online databases (PoWo 2025+; WFO 2025+), allegedly following Hassler (1994–2025), reverted to Christensen’s earlier (1905–1906) assessment but, inexplicably disregarding the principle of priority, accepted *Ctenitis grisebachii* (Baker) Ching in preference to its listed synonym *Aspidium ameristoneuron* Fée.

**Figure 1.**
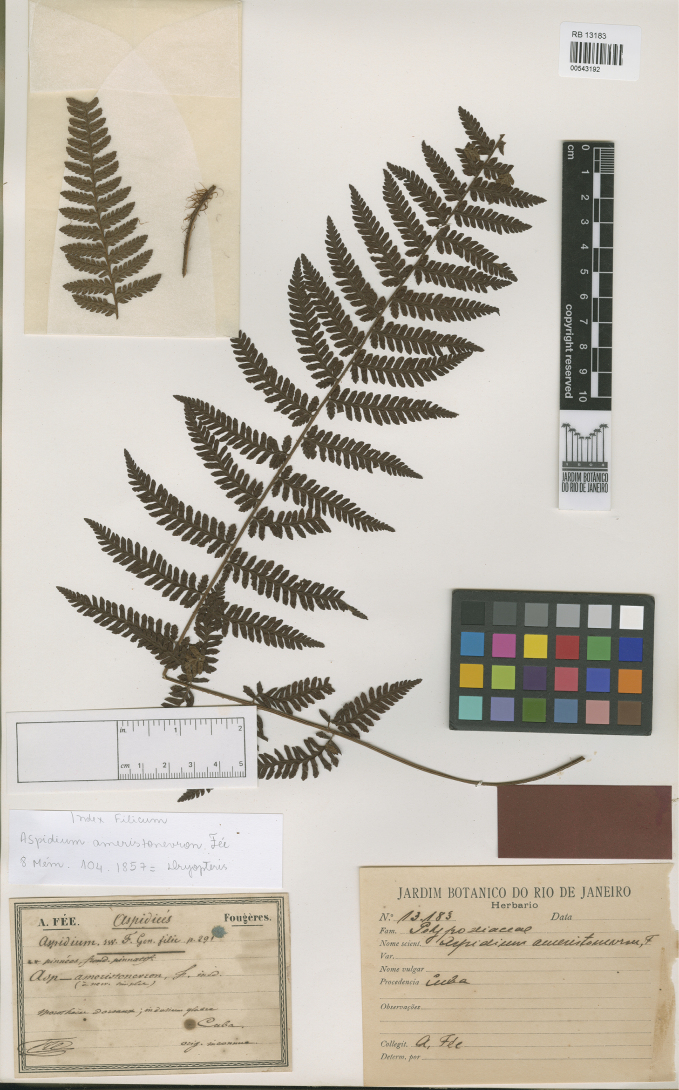
The holotype of *Aspidium ameristoneuron* Fée (RB #543192). Provided and reproduced by kind permission of the curator, Clarice Martins Ribeiro, of the Herbário Dimitri Sucre, Jardim Botânico do Rio de Janeiro.

**Figure 2.**
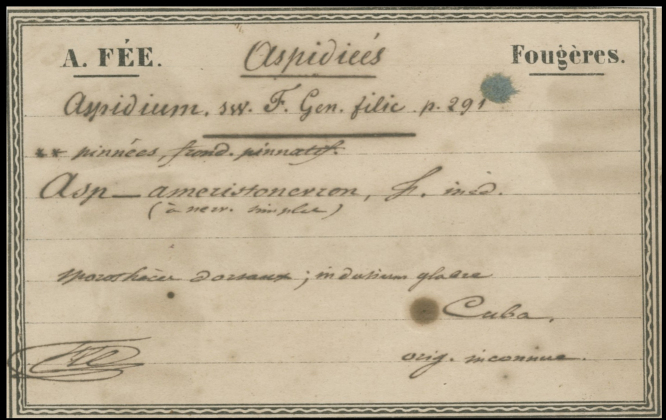
Fée’s label of the holotype specimen (from Fig. 1).

The root of the whole problem lies not only in the scant protologue data on the original material of *Aspidium ameristoneuron* and in the doubt subsequently expressed about its actual provenance but also in the fact that in the Paris herbarium (P)—that one usually considers first when looking for Fée’s material—there is no specimen to be found that would qualify as a potential type. However, as mentioned by Stafleu and Cowan (1976: 818), an important and perhaps the primary part of the Fée herbarium is extant in Rio de Janeiro, where it is now part of the RB collections. Examining the digitized specimen images available in JSTOR’s Global Plants Database, we found a specimen in RB that is, beyond doubt, original material for *Aspidium ameristoneuron* and that, in agreement with Windisch (1982), we consider the holotype of that name. The specimen in question (Fig. 1), best identified through its barcode RB00543192, bears a Fée label (Fig. 2) with the following text elements, in Fée’s unmistakable handwriting:

“*Asp*[*idium*]. *ameristonevron*, F[ée] ined.” “orig[ine]. inconnue” and “Cuba.” The plant itself is definitely not *Ctenitis grisebachii*, nor does it belong to any fern species known from the island of Cuba. The question therefore remains: where does it come from, and to which currently known species does it belong?

## ﻿Results

The key to answering these questions is provided by a note already known to Christensen (1920: 44) and quoted by him in the following terms: “On a label in herb. Mett. (B) Mettenius has written: ‘Aspidium ameristoneuron Fée ined. Mexico. Tabasco. 1489. Jurgensen.’”

**Figure 3.**
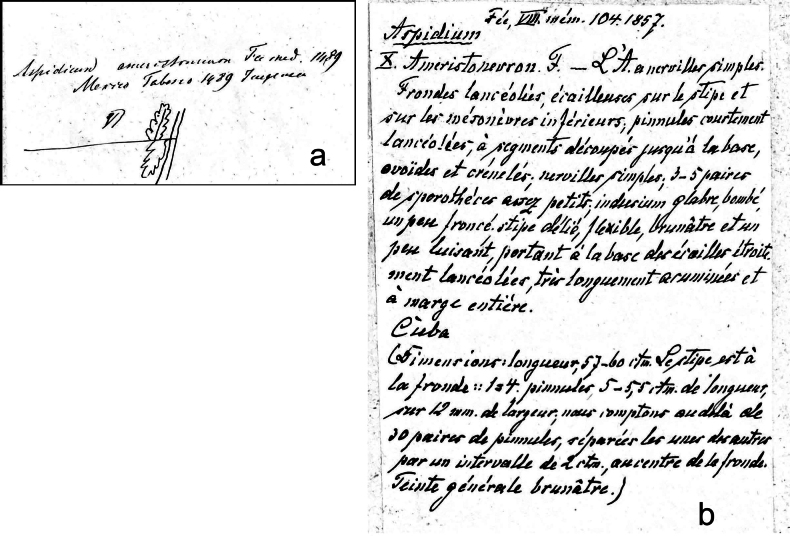
a. Label, without specimen, in Mettenius’s handwriting, in Berlin (B), which is obviously a copy of the label of the specimen Linden 1489 (B #200058730) in Mettenius’s herbarium; it is presumably the missing label of the holotype of *Aspidium ameristoneuron* Fée (RB #543192). b. Fée’s descriptive notes of his new *Aspidium ameristoneuron*, presumably sent to Mettenius upon receipt of the (prospective) holotype specimen. Currently glued to the same sheet as the label in Fig. 3a.

This very label (Fig. 3a) still exists in the Berlin herbarium, where it is glued to a sheet with no plants on it. It was associated in the same folder with two other sheets from the Kuhn or Mettenius herbarium, both collected by C. Wright “in Cuba orientali, Sept. 1859–Jan. 1860” and distributed as No. 1055. One of these was identified by Mettenius as “*Aspidium tuberculatum*,” a name that apparently has never been published, and both are parts of the original material for *Nephrodium grisebachii* Baker (*Ctenitis grisebachii* (Baker) Ching), a widespread Neotropical species that is indeed present in eastern Cuba.

Christensen (l.c.) correctly concluded “that Mettenius’ note is right, that the species of Fée is really Mexican.” Unfortunately, Christensen misread a (to us) illegible word on the label as “Jurgensen,” treating it as the collector’s name (when in fact no collector so named is known to have visited the New World in the 19^th^ century). Otherwise, Christensen would doubtless have concluded that the specimen associated with the label was none other than a duplicate from the gathering Linden 1489, made at Teapa, Tabasco (Mexico), in January 1840, and thus an isosyntype of *Aspidium lindenii* Kuhn, now treated as a synonym of *Ctenitis salvinii* (Baker) Stolze. Such a conclusion is inescapable, given the identity of provenance (Tabasco, Mexico) and collector’s number (1489), as well as the close similarity of the two species concerned.

Most likely, Mettenius, when preparing to send to Fée a duplicate of Linden 1489 from his own herbarium (keeping for himself one frond, now B #200058730), made a copy of the original label and then forgot to include it when mailing the specimen. He may have mentioned Linden as the collector in an accompanying note. Linden’s main and best-known Neotropical collections being those from Cuba, Fée may then have been misled to infer a Cuban origin of the specimen. His handwritten description (Fig. 3b), glued on the Berlin sheet, is likely Fée’s acknowledgment and identification of the received material. All this is to some extent conjectural but perfectly explains the reported factual evidence.

## ﻿Conclusion

A nomenclatural digest of the two species follows, to clarify unmistakably our conclusion.

### ﻿ Ctenitis grisebachii

Taxon classificationPlantae

(Baker) Ching in Sunyatsenia 5: 250. 1940.

E33EC1EA-AA7E-5235-85D0-EBBCD21087E7

≡ Nephrodium grisebachii Baker in Hooker and Baker, Syn. Fil.: 285. 1867. Lectotype (detailed here): [specimen] in Cuba orientali, [prov. Santiago de Cuba, “prope La Guinea”, 14-XII-1859 (according to label of YU #804)], *Wright 1055* (K ##590310!, 590311! [one specimen mounted on 2 sheets]; isolectotypes?: B ##200052230!–200052231!, BM ##605204–605205 [fotos!], BR ##5798568, 5839421 [fotos!]), GH #21050 [foto!], K ##590312!, 1096164 [foto!], PH #19281 [foto!], S #6-471 [foto!], US #67023 [foto!], YU #802–804 [fotos!]).
≡ Dryopteris grisebachii (Baker) Kuntze, Revis. Gen. Pl. 1–2: 812. 1891.

#### Distribution.

S. Mexico, Central America, Cuba, and Jamaica (PoWo 2025+).

### ﻿ Ctenitis ameristoneuros

Taxon classificationPlantae

(Fée) Greuter & R. Rankin comb. nov.

8066CB15-329A-5C49-BDD0-94BDABEF3BB1

≡ Aspidium ameristoneuron (‘*ameristonevron*’) Fée, Mém. Foug. 8, Ic. Esp. Nouv.: 104. 1857. Holotype: [specimen] “Cuba” (in error), without date and collector [in fact: México, Teapa, Tabasco, Jan. 1840, *Linden 1489*] (RB #543192 [foto!]; isotypes (by implication) are listed below as types of Aspidium lindenii.
≡ Lastrea ameristoneuros (‘*ameristoneura*’) (Fée) T. Moore in Index Fil.: 85. 1858.
≡ Dryopteris ameristoneuros (‘*ameristoneura*’) Fée) C. Chr., Index Filic.: 251. 1905.
= Nephrodium salvinii (‘*Salvini*’) Baker in Hooker and Baker, Syn. Fil.: 274. 1867 ≡ Dryopteris salvinii (Baker) Kuntze, Revis. Gen. Pl. 2: 813. 1891 – Lectotype (specified by Stolze 1977: 43): [specimen] “Guatemala”, *Salvin and Godman* (K #590305 [ex herb. Hooker; foto!]; isolectotype: B #200065533 [fragm.!]). – These are at the same time syntypes (isotypes or paralectotypes) of Aspidium lindenii.
≡ Ctenitis salvinii (Baker) Stolze in Amer. Fern J. 67: 43. 1977.
= Aspidium lindenii (‘Lindeni’) Kuhn in Linnaea 36: 116. 1869 – Lectotype (Mickel and Beitel 1988: 134): [specimen] Mexico, *Linden 1489* (B #200058730! [ex herb. Mettenius, “exacte quadrat / Aspidium decipiens” [ined.]]; isolectotypes: BR #13512019, GH #21115 [“Tabasco, Teapa, rochers humides”, Jan 1840, ex herb. T. Moore; foto!], K #590302 [“rochers humides de Teapa (Tabasco)”, ex herb. Hooker; foto!].
≡ Nephrodium lindenii (Kuhn) Baker in Hooker and Baker, Syn. Fil., ed. 2.: 493. 1874.
≡ Dryopteris lindenii (Kuhn) Kuntze in Revis. Gen. Pl. 1–2: 813. 1891.
≡ Ctenitis lindenii (Kuhn) A. R. Sm. in Proc. Calif. Acad. Sci., ser. 4, 40: 229. 1975.

#### Distribution.

Endémic to SE México (Yucatán to Veracruz and Chiapas) and northern Central América (Belice, Guatemala) (PoWo 2025+, under *Ctenitis salvinii*); absent from the Antilles.

## Supplementary Material

XML Treatment for Ctenitis grisebachii

XML Treatment for Ctenitis ameristoneuros

## ﻿Additional information

### Use of AI

No use of AI was reported.

### Author ORCIDs

Werner Greuter https://orcid.org/0000-0002-8677-7544

Rosa Gloria Rankin Rodríguez https://orcid.org/0000-0002-7328-0395

### Data availability

All of the data that support the findings of this study are available in the main text.
